# Context-dependent roles of resistin-like molecule β in mucosal defense and inflammatory diseases

**DOI:** 10.3389/fimmu.2026.1882146

**Published:** 2026-09-24

**Authors:** Qi Ai, Wen Chen, Wen Chen, Qinhui Tuo

**Affiliations:** 1 School of Basic Medical Sciences, Ningxia Medical University, Yinchuan, China; 2 Department of Gastroenterology, The Second Affiliated Hospital, Hengyang Medical School, University of South China, Hengyang, China; 3 Key Laboratory of Vascular Biology and Translational Medicine, Medical School, Hunan University of Chinese Medicine, Changsha, China; 4 Key Laboratory for Quality Evaluation of Bulk Herbs of Hunan Province, School of Pharmacy, Hunan University of Chinese Medicine, Changsha, China

**Keywords:** host–microbiota interactions, immune regulation, membrane permeabilization, mucosal immunity, resistin-like molecule β

## Abstract

Resistin-like molecule β (RELMβ) is a cysteine-rich secreted protein predominantly produced by intestinal goblet cells and regulated by microbial colonization, epithelial transcriptional programs, and immune signals. At the intestinal mucosal surface, recombinant murine and human RELMβ permeabilize susceptible bacterial membranes, while genetic studies in mice support its role in maintaining spatial separation between the microbiota and the epithelium. Murine studies further implicate RELMβ in defense against selected gastrointestinal nematodes, epithelial repair, intestinal inflammation, and the microbiota-dependent establishment of oral tolerance. Beyond the intestine, RELMβ has been associated with pulmonary vascular and airway remodeling, metabolic liver disease, atherosclerosis, abdominal aortic aneurysm, and selected cancers. However, the strength of this evidence varies: most causal findings derive from rodent genetic or intervention models, whereas human evidence is based largely on tissue associations and cultured-cell experiments. Mechanistically, RELMβ may act through at least two non-exclusive modes: direct membrane disruption and modulation of host-cell responses, but the relationship between these activities remains unclear. Its physiological oligomeric state, context-specific molecular interaction partners, and cellular source in the circulation and extraintestinal lesions also remain unresolved. This review distinguishes direct RELMβ evidence from inferences based on other RELM-family proteins and proposes a compartment–interaction system framework that considers protein source, route of exposure, molecular assembly, local physicochemical conditions, and available interaction partners as potential determinants of biological outcome. This framework is presented as a testable synthesis for defining experimental priorities and assessing the biological and therapeutic relevance of RELMβ.

## 1 Introduction

Resistin-like molecule β (RELMβ) is a cysteine-rich secreted protein produced predominantly by intestinal epithelial goblet cells under physiological conditions (1, 2). This distribution distinguishes it from resistin, which is produced mainly by adipocytes in mice (3) and by monocytes and macrophages in humans (4), and places RELMβ at the interface among the intestinal epithelium, immune system, and gut microbiota. Its expression is regulated by epithelial differentiation, microbial colonization, and type 2 cytokine signaling, allowing RELMβ production to respond to changes in the mucosal environment (1, 2, 5, 6). Human *RETNLB* and murine *Retnlb* are commonly treated as corresponding RELMβ genes; however, lineage-specific expansion of the rodent *Retnl* lineage complicates strict one-to-one functional comparisons among human RELMβ and the murine RELM proteins (7). Mechanisms identified for one RELM-family member should therefore not be assigned to another without direct experimental support (**Figure 1**).

**Figure 1:**
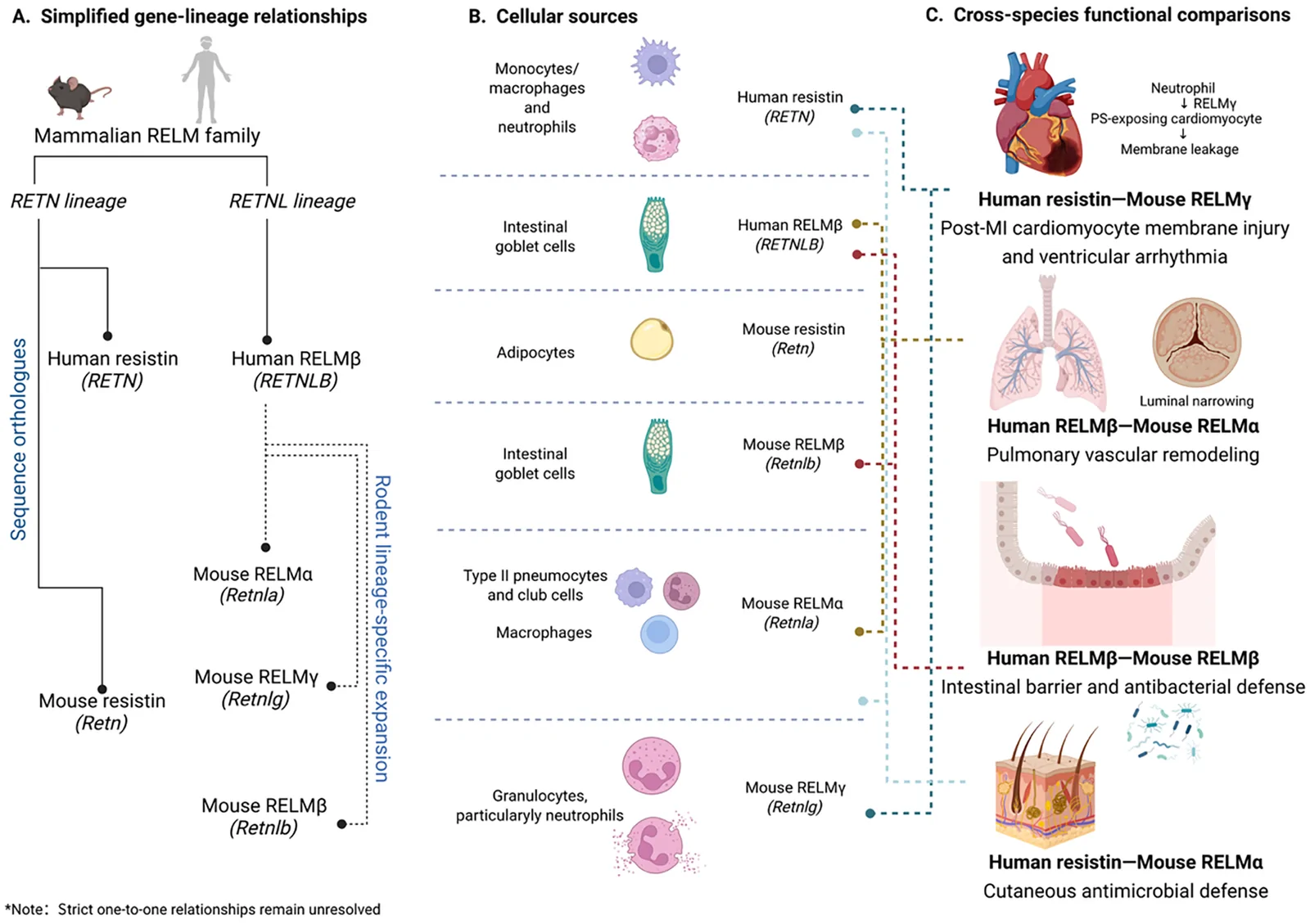
RELM family: gene lineages, cellular sources, and functional comparisons. Simplified relationships between human and murine RELM-family genes, their predominant cellular sources, and reported cross-species functional comparisons. In panel **(A)**, dotted branches indicate rodent lineage-specific expansion; in panel **(B)**, the predominant cellular sources of individual RELM-family members are shown; in panel **(C)**, colored dashed lines connect context-specific functional comparators and do not imply sequence orthology or universal functional equivalence. Created in BioRender. ai, Q. (2026) https://BioRender.com/up91war.

Pore-forming or pore-like membrane activity has emerged as a recurrent property among several RELM-family proteins, with RELMβ providing the best-characterized example at the intestinal mucosal surface. Recombinant murine and human RELMβ permeabilized and killed susceptible Gram-negative bacteria, while studies using murine RELMβ further demonstrated binding to negatively charged membrane lipids and formation of size-selective membrane pores (8). In *Retnlb*-deficient mice, *Proteobacteria* penetrated the normally sterile inner mucus layer and entered mucosal tissues, supporting a role for RELMβ in maintaining spatial separation between the microbiota and the intestinal epithelium (8). RELMβ also contributes to defense against selected lumen-dwelling nematodes by impairing their sensory and feeding functions rather than directly killing them (1, 9). Beyond direct host defense, RELMβ regulates epithelial and immune responses during intestinal inflammation (10–14) and participates in the microbiota-dependent establishment of oral tolerance during the peri-weaning period (15). RELMβ has also been associated with pulmonary vascular remodeling (16–23), metabolic liver disease (24), atherosclerosis (25), and abdominal aortic aneurysm (26). The strength of this evidence varies substantially: most causal findings derive from rodent genetic or intervention models, whereas human evidence consists mainly of tissue associations and cultured-cell experiments. Some proposed mechanisms are also inferred from other RELM-family members and cannot yet be attributed directly to human RELMβ.

Previous reviews have summarized the broad signaling pathways and disease associations of the RELM family, but RELMβ-specific evidence has not been consistently separated from cross-species and cross-family mechanistic inference (27, 28). Current evidence indicates that RELMβ can act through at least two non-exclusive modes: direct interaction with biological membranes (8) and modulation of host-cell responses (16, 18, 19, 22, 29–31). How these activities are related remains unclear. Its physiological oligomeric state (8, 32), context-specific molecular interaction partners (16, 33, 34), and cellular source in the circulation and extraintestinal lesions (17, 25, 35–37) also remain unresolved. In particular, it is not known whether RELMβ detected outside the intestine is produced locally, released basolaterally by intestinal epithelial cells, or redistributed following epithelial barrier disruption. This review examines RELMβ expression, structure, membrane activity, immune regulation, and involvement in disease while distinguishing evidence by species, RELM-family member, and experimental system. We further propose a compartment–interaction system framework that considers cellular source, route of exposure, molecular assembly, local physicochemical environment, and available interaction partners as potential determinants of biological outcome. These observations raise three related questions: which molecular interaction systems operate in different tissues, how receptor-mediated responses relate to direct membrane activity, and where circulating or lesion-associated RELMβ originates.

## 2 Molecular biology, expression, and regulation of RELMβ

### 2.1 RELM gene and protein family

Comparative genomic analyses distinguish two evolutionarily related branches within the mammalian RELM family: the ancestral *Retn* lineage and the derived *Retnl* lineage (7). RELM family proteins are widely conserved across mammals; however, the number of encoded genes and their expression patterns differ substantially among species (38). These differences should be considered when interpreting findings derived from rodent models. The mouse genome encodes four members: RELMα (*Retnla*), RELMβ (*Retnlb)*, RELMγ (*Retnlg*), and resistin (*Retn*) (27). By contrast, the human genome encodes two paralogous RELM-family proteins, RELMβ (*RETNLB*) and resistin (*RETN*) (27). In mice, *Retnla*, *Retnlb* and *Retnlg* are clustered on chromosome 16, while *Retn* is located on chromosome 8; human *RETNLB* and *RETN* map to chromosomes 3 and 19, respectively (27, 28). Lineage-specific expansion of the rodent *Retnl* locus complicates assignment of a strict one-to-one relationship between human *RETNLB* and any individual murine *Retnl* gene (7).

Evolutionary relationships should also be distinguished from experimentally defined functional correspondence. Although human *RETN* is the sequence orthologue of murine *Retn (*7, 39), human resistin has been used as a functional comparator for different murine RELM proteins in different experimental settings. Context-specific functional parallels include human resistin and murine RELMα in cutaneous antimicrobial defense (40), helminth infection (41), and pulmonary hypertension-associated right-ventricular dysfunction and remodeling (42); human RELMβ and murine RELMα in pulmonary vascular remodeling (43, 44); human resistin and murine RELMβ in macrophage activation (10, 45) and bactericidal membrane permeabilization (8); and human resistin and murine RELMγ in ischemia-associated cardiomyocyte membrane injury (46). Such comparisons may reflect similarities in cellular source, biochemical activity, or disease-associated phenotype, but they do not establish a universal one-to-one functional equivalence between human resistin and any individual murine RELM protein. Consequently, the relevant murine comparator should be specified according to the tissue and biological process under investigation rather than inferred solely from gene nomenclature (**Figure 1**; **Table 1**).

**Table 1:** Selected evolutionary and context-specific experimental relationships between human and murine RELM-family proteins.

Human protein and gene	Murine protein and gene	Relationship type	Biological context	Relevant source or target cells	Evidence-supported comparison	References
Human resistin (*RETN*; 19p13.2)	Murine resistin (*Retn*; chromosome 8)	Sequence orthologues	Metabolic and inflammatory biology	Human monocytes/macrophages; murine adipocytes	Although the genes are orthologous, their predominant cellular sources and reported biological roles differ substantially between species.	(3, 4, 7, 39)
Human RELMβ (*RETNLB*; 3q13.13)	Murine RELMβ (*Retnlb*; chromosome 16B5)	Orthologous RELMβ pair	Intestinal antimicrobial defense	Intestinal goblet cells; bacterial membranes	Recombinant proteins from both species show bactericidal activity, whereas lipid binding, membrane-associated multimerization, and size-selective permeabilization were characterized mainly for murine RELMβ.	(8)
Human RELMβ (*RETNLB*; 3q13.13)	Murine RELMα/HIMF (*Retnla*; chromosome 16B5)	Context-specific functional comparator	Pulmonary hypertension	Pulmonary vascular cells	HIMF promotes pulmonary vascular remodeling in rodent models; related BMPR1A–BMPR2 effects of human RELMβ have been examined mainly in cultured human pulmonary artery smooth muscle cells.	(16)
Human resistin (*RETN*; 19p13.2)	Murine RELMβ (*Retnlb*; chromosome 16B5)	Context-specific functional comparator	Macrophage activation and bacterial membrane permeabilization	Macrophages; bacterial or model membranes	Similar inflammatory and membrane-active properties have been reported, but these observations do not establish evolutionary or universal mechanistic equivalence.	(8, 10, 45)
Human resistin (*RETN*; 19p13.2)	Murine RELMγ (*Retnlg*; chromosome 16B5)	Family-based membrane-activity comparator	Myocardial ischemia and ventricular arrhythmia	Neutrophils; injured cardiomyocytes	Murine RELMγ and human resistin produced pore-like membrane defects in experimental systems; equivalent pathogenic membrane activity of human resistin in patients has not been established.	(46)
Human resistin (*RETN*; 19p13.2)	Murine RELMα (*Retnla*; chromosome 16B5)	Context-specific functional comparator; not orthologues	Cutaneous antimicrobial defense	Keratinocytes, sebocytes, and bacterial membranes	Murine RELMα and human resistin have been compared for direct antimicrobial membrane activity, but murine RELMα has no direct human orthologue.	(40)

Because RELM-family proteins share conserved sequence features and some available antibodies cross-react across isoforms or species, expression and localization data should be interpreted using isoform- and species-specific validation and, where possible, genetic controls (47).

### 2.2 Structure of RELMβ

Human RELMβ is encoded by *RETNLB*, which maps to chromosome 3q13.13, and is synthesized as a 111-amino-acid precursor containing an N-terminal signal peptide. Its murine counterpart, *Retnlb*, encodes a 105-amino-acid precursor (39, 48). The canonical human and murine precursor sequences share approximately 56% amino acid identity, with greater conservation in the cysteine-rich C-terminal region (47, 48). Despite their orthologous relationship and C-terminal sequence conservation, these features do not by themselves establish mechanistic equivalence across species.

Available structural evidence derives primarily from the X-ray crystal structure of recombinant murine RELMβ (32). Following removal of the signal peptide, the mature murine RELMβ protomer comprises two structural regions: (i) an N-terminal α-helical tail, which participates in three-stranded coiled-coil assembly; and (ii) a cysteine-rich C-terminal β-sandwich head domain containing 10 conserved cysteine residues that form five intramolecular disulfide bonds within each protomer and stabilize the fold (32). In addition, the three Cys2 residues in each trimer, numbered according to the mature murine RELMβ sequence, collectively form three intertrimer disulfide bonds with the corresponding residues in the opposing trimer, linking the two trimers in a tail-to-tail orientation to produce a hexameric assembly in the crystal structure (32) (**Figure 2**).

**Figure 2:**
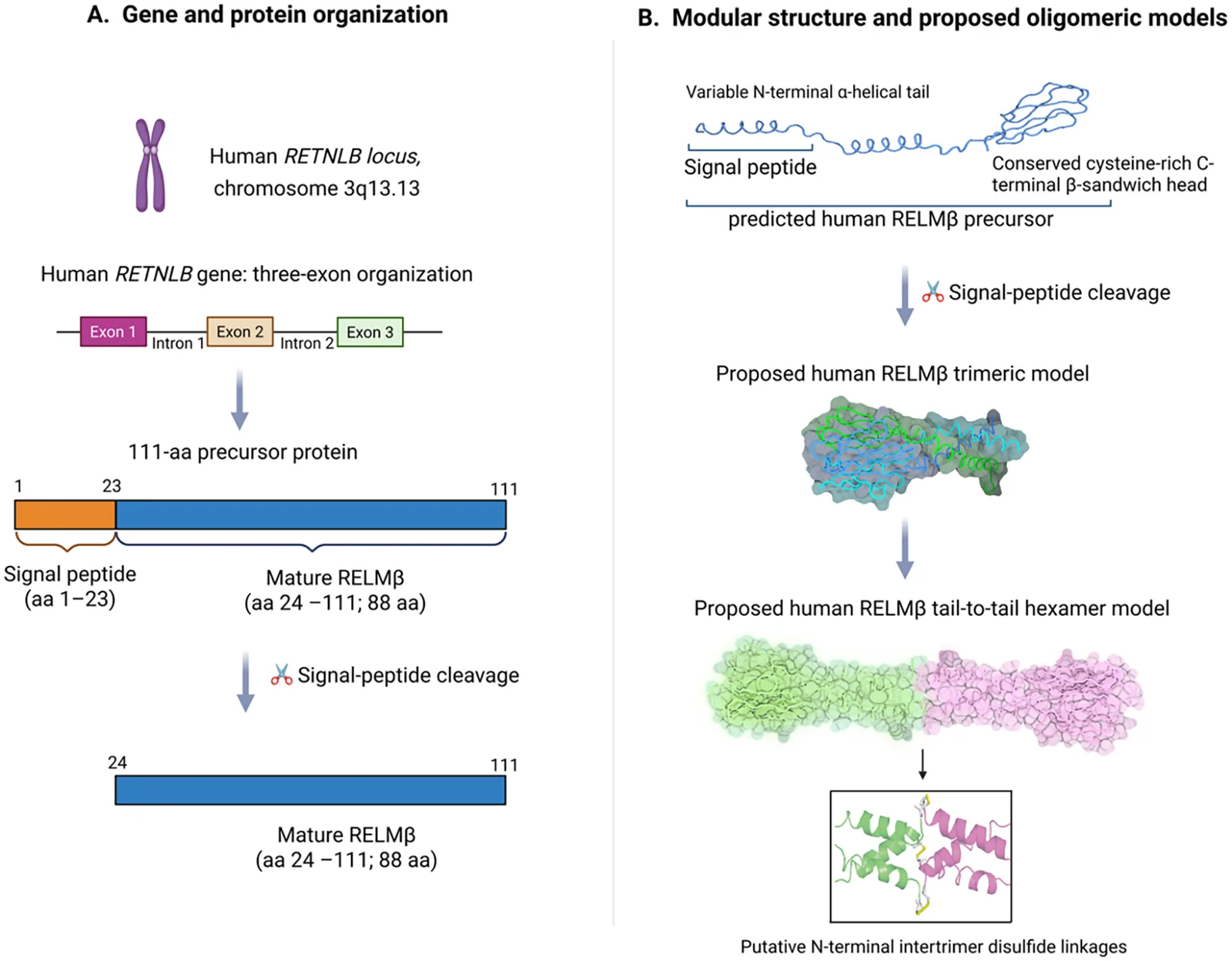
Human RELMβ gene and protein organization and proposed oligomeric models. **(A)** The human *RETNLB* locus, three-exon organization, 111-amino-acid precursor, and predicted signal-peptide cleavage yielding an 88-amino-acid mature protein. **(B)** Predicted modular organization of the RELMβ precursor and structural models of trimeric and tail-to-tail hexameric assemblies. The intertrimer disulfide linkages and oligomeric arrangements are proposed models; the predominant physiological assembly state of human RELMβ remains unresolved. Created in BioRender. ai, Q. (2026) https://BioRender.com/d6be2yu.

Biochemical and crystallographic studies of recombinant murine RELMβ have identified apparent disulfide-linked dimers as well as coiled-coil trimers assembled into disulfide-linked hexamers (32, 49). Because these forms were detected using different protein preparations and analytical methods, their structural relationship and relative prevalence *in vivo* remain uncertain. RELMβ may therefore adopt multiple assembly states, but no single state has been shown to predominate physiologically or to be universally required for its biological activity (see Section 2.3).

### 2.3 Structure-function relationship of RELMβ

Although crystallographic and biochemical studies have defined an oligomeric architecture for recombinant RELMβ, its physiological assembly states and receptor-recognition mechanisms remain incompletely understood. Resistin provides a structurally related comparator; however, mechanisms established for resistin cannot be directly assigned to RELMβ. Murine resistin assembles into coiled-coil trimers and disulfide-linked tail-to-tail hexamers; analysis of an intertrimer disulfide-deficient Cys6Ser mutant suggests that these disulfide bonds regulate the balance between higher- and lower-order oligomeric states, which may differ in biological activity (32). In human monocytes, resistin was shown to bind directly to CAP1, implicating the central proline-rich SH3-binding region of CAP1 in ligand recognition (45). Subsequent work suggested that the resistin-CAP1 complex also operates in obesity-associated skeletal-muscle mitochondrial dysfunction (50). A recent study systematically demonstrated that rodent HIMF/RELMα binds the DTLPF motif in the extracellular domain of BMPR1A and disrupts the BMPR1A–BMPR2 complex (16). The authors further examined human RELMβ as a putative functional analogue of rodent HIMF and reported similar effects in human pulmonary artery smooth muscle cells. However, because human RELMβ and rodent RELMα are paralogous family members rather than true orthologues, their mechanistic equivalence remains uncertain and requires direct validation.

RELM-family precursors share a modular organization comprising an N-terminal signal peptide and, in the mature protein, a variable N-terminal α-helical tail and a conserved cysteine-rich C-terminal β-sandwich head (32, 48, 49). Available evidence suggests that these regions make distinct but context-dependent contributions to function (see Section 6.2). Crystallographic analysis of recombinant murine RELMβ expressed in HEK293T cells showed that the N-terminal α-helical tails assemble into three-stranded coiled coils and contribute to higher-order oligomerization (32). By contrast, the recombinant proteins used in the antibacterial and membrane-based assays described below, including murine and human RELMβ, were produced in *Escherichia coli* and purified as folded monomers (8). In membrane-based assays, the isolated C-terminal region of murine RELMβ, but not its N-terminal region, mediated binding to negatively charged phospholipid membranes and liposome permeabilization, while retaining membrane-associated multimerization (8). In the bacterial permeabilization assays, recombinant murine RELMβ was tested at 5 μM, with maximal activity observed at pH 5.5 and 25 mM NaCl; liposome dye-release assays used 5 μM protein and 100 μM phosphatidylcholine/phosphatidylserine (PC/PS) liposomes (8). Following cross-linking of purified full-length murine RELMβ in the presence of liposomes, the products were solubilized in detergent and separated by size-exclusion chromatography; immunoblotting detected an approximately 60–70-kDa species, consistent with an estimated assembly of six to eight subunits (8). However, the precise architecture of this complex was not resolved, and the membrane-associated oligomeric state of human RELMβ was not similarly characterized. Murine RELMβ-mediated antibacterial and membrane-permeabilizing activities were enhanced under acidic, low-ionic-strength conditions, indicating that the physicochemical environment modulates its membrane-disruptive function (8).

Thus, a C-terminal effector function has been directly demonstrated for the antibacterial membrane activity of recombinant murine RELMβ. By contrast, the region of RELMβ responsible for host-cell receptor recognition and receptor-dependent signaling has not been defined. The C-terminal membrane activity established in the antibacterial setting should not be extrapolated to receptor-mediated actions of RELMβ in pulmonary hypertension or other host-cell disease contexts.

### 2.4 Regulation of RELMβ expression

Under physiological conditions, RELMβ expression is concentrated in intestinal epithelial goblet cells (1, 48). CDX2 contributes to basal and intestine-associated RELMβ transcription (2). The epithelial transcriptional program is further shaped by HNF4α isoforms: P2-HNF4α binds and preferentially activates the RELMβ promoter compared with P1-HNF4α, providing an additional isoform-specific mechanism linking epithelial state to RELMβ expression (51). Commensal colonization is another major determinant: intestinal RELMβ expression and secretion are markedly reduced in germ-free mice and rapidly restored following conventionalization (5). This microbiota dependence was independently supported by the observation that colonic *Retnlb* expression is nearly undetectable in germ-free mice and progressively induced during postnatal microbial colonization (52). Conversely, transplantation of microbiota from protist-exposed human donors reduced small-intestinal *Retnlb* expression in germ-free mice, further indicating that microbiota-dependent regulation varies with community composition (53). More recently, monoassociation of germ-free mice with *Enterocloster clostridioformis* selectively increased *Retnlb* expression in cecal epithelial cells and was associated with reduced *Salmonella Typhimurium*-induced pathology, indicating that RELMβ regulation may depend on specific members of the microbiota rather than microbial colonization alone (54). Notably, bacterial colonization did not measurably alter colonic *CDX2* mRNA abundance, protein distribution, or phosphorylation, while nematode infection induced RELM-family expression without altering *Cdx2* expression (2). Thus, epithelial differentiation establishes basal expression competence, whereas environmental and immune signals provide additional inducible regulation.

Type 2 cytokine signaling is the best-characterized inducible pathway. IL-4 and IL-13 increase *Retnlb* expression through IL-4Rα–dependent signaling, with an additional contribution from PI3K–AKT, whereas IFN-γ suppresses RELMβ expression (1, 6, 55). Accordingly, RELMβ is strongly induced during helminth infection and selected enteric bacterial infections (1, 9, 11). Recent studies using mouse and human intestinal organoids further showed that exogenous RELMβ increases endogenous *Retnlb/RETNLB* expression and cooperates with IL-13, supporting the existence of autoregulatory and type 2 cytokine-dependent amplification loops (15). Other signals may also modulate expression: TNF-α, insulin, and stearic acid increased *RETNLB* expression in LS174T cells, whereas glucose reduced it, indicating that nutrient- and inflammatory-signal effects are context dependent (56).

Outside the intestine, allergens and IL-4/IL-13 induce pulmonary *Retnlb* expression largely through STAT6-dependent signaling in mice (55), while hypoxia induces human *RETNLB* expression in cultured pulmonary epithelial and vascular cells (57). Constitutive STAT6 activation in murine Club cells induced pulmonary expression of RELMα, RELMβ, and RELMγ together with mucus secretion, but was insufficient to alter the outcomes of helminth infection, fungal-allergen challenge, or influenza-virus infection (58). TGF-β1 also directly increases *RETNLB* transcription through SMAD2/3/4 in human endothelial cells (29). Dietary and metabolic regulation is more variable: increased colonic expression and circulating RELMβ concentrations have been reported in high-fat-diet-fed and *db/db* mice (35), whereas high-protein and high-carbohydrate diets suppressed intestinal *Retnlb* expression, and another high-fat/high-cholesterol dietary model reduced colonic *Retnlb* transcripts and RELMβ protein (56, 59). More recently, dietary pectin increased small-intestinal RELMβ expression in mice through a tuft cell–ILC2–IL-13–STAT6 signaling axis, providing an additional mechanism by which dietary components indirectly regulate epithelial RELMβ production (60).

Overall, RELMβ expression is governed by the interaction of epithelial identity, microbial exposure, cytokine signaling, and the local metabolic environment, with the relative contribution of each pathway depending on species, tissue, and disease context.

### 2.5 Secretion and extracellular fate of RELMβ

RELMβ is synthesized as a secreted protein and is released predominantly by intestinal goblet cells into the mucus layer and intestinal lumen, where it contributes to spatial separation between the microbiota and the epithelial surface (1, 5, 8). Cathelicidin-dependent secretion has also been reported. Murine CRAMP was required for normal goblet-cell mucus secretion during *Citrobacter rodentium* infection, whereas human LL-37 stimulated the reactive oxygen species-dependent release of RELMβ and TFF3 from LS174T goblet-like cells (61).

As discussed in Section 2.3, RELMβ can form disulfide-linked dimers and higher-order assemblies (8, 32, 49). Membrane-associated multimerization has been observed in liposome assays, although the distribution and stability of these assemblies under physiological conditions remain incompletely characterized. In particular, the oligomeric state of RELMβ in the intestinal lumen and human circulation has not been resolved systematically.

In one study, measurable plasma RELMβ concentrations of approximately 0.087–0.167 ng/mL were reported in about half of the participants (36), although many measurements were close to the assay’s limit of quantification. Circulating RELMβ was also increased in patients and mouse models of food allergy (15, 62). It remains unclear whether circulating RELMβ results from regulated basolateral secretion by intestinal epithelial cells, passage across a disrupted epithelial barrier, or production by extraintestinal cells. Its tissue distribution, clearance pathways, systemic half-life, and physiologically relevant circulating oligomeric state remain largely unknown.

## 3 RELMβ in mucosal immunity and homeostasis

### 3.1 Host defense

RELMβ functions as a secreted effector at the intestinal mucosal surface. Recombinant murine and human RELMβ killed the Gram-negative bacteria tested, including *Citrobacter rodentium* and *Pseudomonas aeruginosa*. Studies using murine RELMβ further demonstrated binding to negatively charged membrane lipids and assembly into size-selective, membrane-permeabilizing pores (8). In *Retnlb*^−/−^ mice, Proteobacteria penetrated the inner colonic mucus layer and associated with the mucosal tissue, whereas the total luminal bacterial burden was not significantly altered (8). These findings support a role for RELMβ in maintaining spatial separation between the microbiota and the epithelium rather than broadly controlling intestinal bacterial abundance.

RELMβ also contributes to defense against selected gastrointestinal nematodes, although its importance depends on the parasite and its anatomical niche. Initial studies showed that RELMβ binds nematode chemosensory structures and inhibits chemotaxis *in vitro (*1). Subsequent genetic experiments demonstrated that RELMβ contributes to the spontaneous and IL-4-induced expulsion of the lumen-dwelling nematodes *Nippostrongylus brasiliensis* and *Heligmosomoides polygyrus*. Rather than directly killing the worms, RELMβ impaired their ability to feed, resulting in reduced ATP content and fecundity. By contrast, RELMβ was dispensable for immunity against *Trichinella spiralis*, which occupies an intraepithelial niche (9). Later studies further indicated that the effects of RELMβ during *N. brasiliensis* infection occur alongside the distinct and partly dominant immunoregulatory effects of RELMα (63, 64). These findings indicate that RELMβ is not a universal anti-helminth effector but preferentially acts against parasites exposed to intestinal luminal secretions (65).

More recently, HpBoRB, a protein containing two complement control protein domains, was identified in *H. polygyrus bakeri* and shown to bind murine RELMβ with subnanomolar affinity (66), but it is a parasite-derived binding protein rather than a host receptor. Transcripts encoding HpBoRB were most abundant during the early, tissue-dwelling L4 stage and declined markedly thereafter, while the protein was detected in L4 larvae but not in adult excretory–secretory products. This interaction raises the possibility that HpBoRB acts as a parasite countermeasure against RELMβ-mediated defense. However, whether HpBoRB and RELMβ encounter each other *in vivo* and whether HpBoRB alters RELMβ activity or parasite fitness have not been established.

Overall, RELMβ acts directly against susceptible bacteria and selected gastrointestinal nematodes, but its effects depend on the target organism and, for helminths, the parasite niche.

### 3.2 Immune regulation

Beyond its direct antimicrobial and anti-helminth activities, RELMβ regulates interactions between intestinal epithelial and immune cells. In chronic low-dose *Trichuris muris* infection, *Retnlb* deficiency reduced parasite-specific CD4^+^ T-cell production of IFN-γ and TNF-α and attenuated intestinal inflammation. *In vitro*, recombinant RELMβ increased macrophage expression of MHC class II and production of IL-12/23p40, thereby enhancing antigen-specific IFN-γ production by CD4^+^ T cells (30). These findings suggest that RELMβ can promote macrophage–T-cell communication and amplify type 1 inflammation in a chronic infection setting.

RELMβ can also support protective immune responses during bacterial infection. In *Citrobacter rodentium*-infected mice, *Retnlb* deficiency delayed intestinal recruitment of CD4^+^ T cells and was associated with reduced IL-22 production, impaired epithelial proliferation, and greater mucosal injury. Administration of recombinant RELMβ restored CD4^+^ T-cell recruitment and increased IL-22 levels and epithelial proliferation (11). RELMβ did not directly stimulate epithelial proliferation, indicating that its protective effect was mediated primarily through immune–epithelial interactions.

More recently, RELMβ was identified as a microbiome-dependent regulator of oral tolerance. In food-allergy-prone mice, epithelial or secretory-cell-specific deletion of *Retnlb* reduced food-antigen-specific IgE responses and protected against anaphylaxis. Mechanistically, RELMβ depleted indole-producing *Lactobacillus* and *Alistipes* populations, thereby reducing microbial indole metabolites that activate the aryl hydrocarbon receptor and support food-allergy-protective RORγt^+^ regulatory T cells. RELMβ antagonism during the peri-weaning period restored this tolerogenic pathway and provided sustained protection against food allergy in susceptible mice (15). Elevated circulating RELMβ was also observed in patients with food allergy, although its causal role in humans remains to be established (15).

Collectively, RELMβ connects epithelial responses with adaptive immunity, but its consequences vary by context: it can support infection-induced tissue repair, amplify chronic inflammatory responses, or impair tolerance to dietary antigens.

### 3.3 Mucosal homeostasis

Studies in *Retnlb*-deficient mice support a role for RELMβ in basal colonic barrier function. These mice showed increased epithelial permeability and reduced colonic expression of the antimicrobial lectins Reg3β and Reg3γ, although they did not develop overt spontaneous intestinal disease (12). Recombinant RELMβ also increased *MUC2* expression and secretion in human mucus-secreting epithelial cells and stimulated *Muc2* secretion from murine intestinal goblet cells *in vivo (*13). Together with its ability to restrict bacterial contact with the colonic mucosa (8), these findings suggest that RELMβ contributes to barrier maintenance through several mechanisms, although the relative importance of mucus secretion, antimicrobial lectin regulation, and direct bacterial killing remains unresolved.

Single-cell transcriptomic analyses have identified *Retnlb* as a highly enriched gene in Reg4^+^ deep crypt secretory cells. Exogenous RELMβ increased *Muc2* expression in mouse colonoids, suggesting a possible role in regulating epithelial secretory function (67). DCS cells themselves support the colonic stem-cell niche, but current evidence does not establish that RELMβ mediates this function. The specific contribution of DCS-derived RELMβ to epithelial maintenance therefore remains to be determined.

The effects of RELMβ are altered when mucus-barrier integrity is compromised. In *Muc2*^−/−^ mice, RELMβ promoted epithelial RegIIIβ expression, depletion of colonic *Lactobacillus* species, and spontaneous colitis, whereas additional deletion of *Retnlb* attenuated microbial dysbiosis and disease severity (14). Age may provide another relevant context. In one study, colonic *Retnlb* expression was approximately 80% lower in 25-month-old than in 2-month-old mice and correlated with reduced expression of several other antimicrobial genes and increased abundance of Gram-negative bacterial taxa (68). These associations do not establish RELMβ loss as a cause of age-related dysbiosis or systemic inflammation. Thus, RELMβ participates in colonic barrier and microbial homeostasis, but its effects depend on the integrity of the mucus layer and the surrounding physiological context.

## 4 Role in chronic inflammatory diseases

The protective role of RELMβ in acute host defense contrasts sharply with its reported role in chronic inflammatory diseases. In the context of chronic diseases, sustained or dysregulated RELMβ expression may shift its predominant activity from physiological defense toward inflammation and tissue injury. This context-dependent functional shift illustrates how a molecule that is beneficial in host defense may become detrimental under specific pathological conditions, although the underlying mechanisms remain incompletely defined (**Figure 3**).

**Figure 3:**
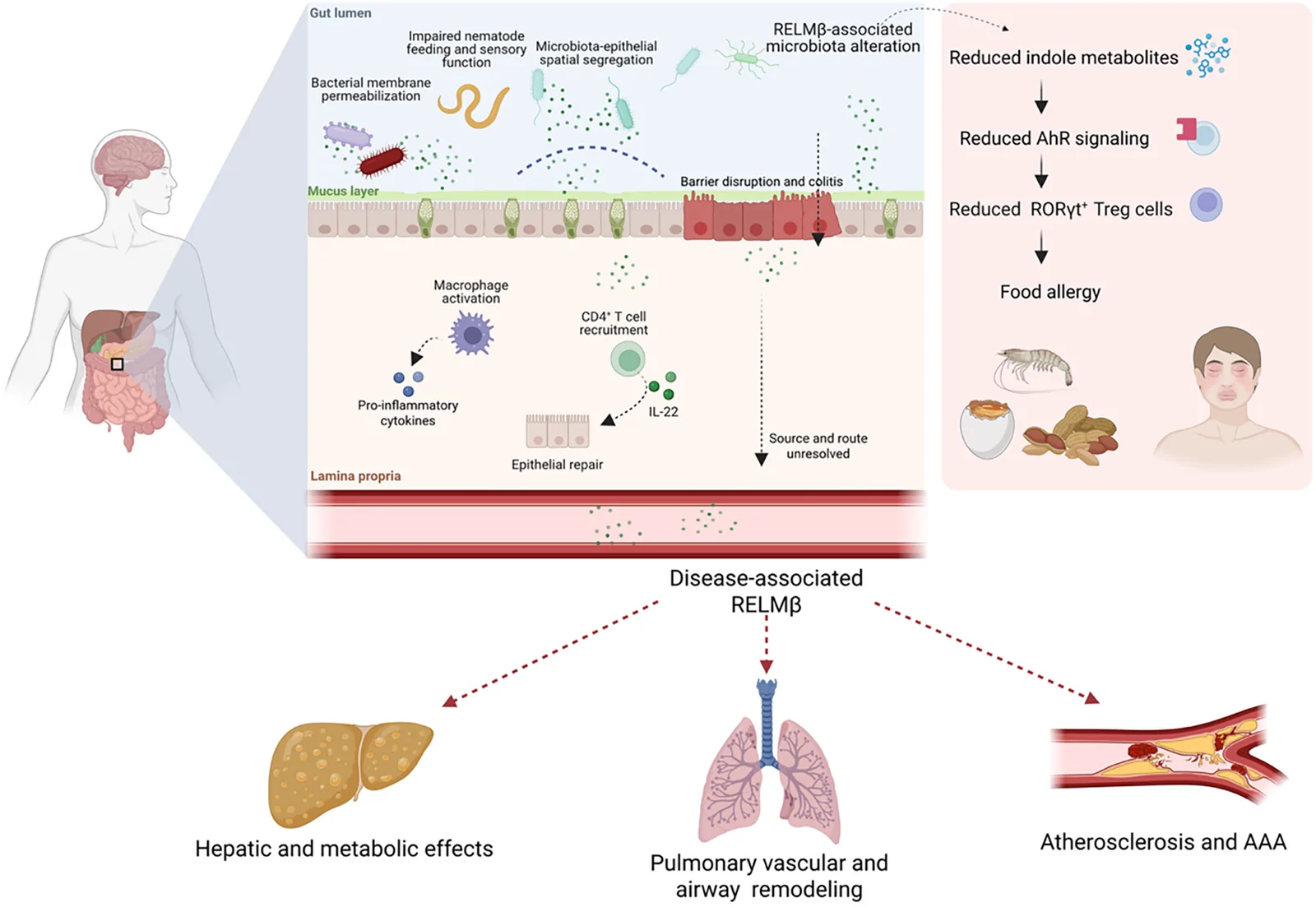
Compartment-dependent functions of RELMβ. Luminal RELMβ contributes to antimicrobial defense, nematode impairment, and microbiota–epithelial segregation, whereas exposure within the lamina propria is associated with immune activation and epithelial repair. Microbiota-dependent RELMβ activity may impair food tolerance, and disease-associated RELMβ has been linked to hepatic, pulmonary, and vascular pathology. Dashed arrows indicate proposed or unresolved routes of extraintestinal RELMβ exposure and do not establish a compartment-driven functional switch. AAA, abdominal aortic aneurysm; AhR, aryl hydrocarbon receptor. Created in BioRender. ai, Q. (2026) https://BioRender.com/b8aswuy.

### 4.1 Intestinal inflammation

Available studies do not support a uniformly protective or pathogenic role for RELMβ in intestinal inflammation. Instead, murine RELMβ produced different outcomes across models of mucosal defense, chemical injury, and mucus-barrier deficiency. Most mechanistic and causal evidence comes from mice, whereas evidence for human RELMβ is largely limited to recombinant-protein experiments and intestinal epithelial cell lines. The evidentiary basis and limitations of the principal disease studies are summarized in **Table 2**.

**Table 2:** Evidence stratification for disease-associated RELMβ and related RELM-family proteins.

Species and RELM member	Disease context	RELMβ source and relevant cell type or tissue	Experimental system	Receptor, pathway, or proposed mechanism	Evidence-supported outcome and limitation	Evidence category and strength	References
Mouse RELMβ	DSS-induced colitis	Bacterially induced RELMβ is produced predominantly by colonic goblet cells; its passage into the lamina propria after epithelial injury was not directly demonstrated	DSS-treated wild-type and *Retnlb*^−/−^ mice; macrophage assays	Proinflammatory cytokine induction, including TNF-α, IL-15	*Retnlb* deficiency reduced DSS-induced injury, supporting a pathogenic role in this chemically disrupted-barrier model; applicability to human IBD remains uncertain	Direct causal evidence in mice: moderate and model-specific	(10)
Mouse RELMβ	TNBS-induced colitis	Goblet-cell-derived RELMβ acts within the intestinal mucus compartment	TNBS-induced colitis and recombinant RELMβ administration	Increased mucin expression and mucus secretion	Recombinant RELMβ attenuated TNBS colitis, indicating a model-specific protective effect associated with mucus-barrier reinforcement	Direct interventional evidence in mice: limited to moderate; recombinant-protein dependent	(13)
Mouse RELMβ	Spontaneous colitis in *Muc2*-deficient mice	RELMβ is markedly increased in the colon and acts at the epithelial–microbiota interface	*Muc2*^−/−^ and *Muc2*^−/−^*Retnlb*^−/−^ mice	RegIIIβ induction, microbial dysbiosis, and depletion of protective *Lactobacillus* species	Genetic loss of RELMβ reduced spontaneous colitis in this model, supporting a microbiota-dependent pathogenic effect under severe mucus-barrier deficiency	Direct genetic evidence: moderate to strong within this model	(14)
Mouse RELMβ	SAMP1/YitFc ileitis	RELMβ is highly expressed in the ileum during early disease	SAMP1/YitFc mice	Association with TNF-α, IL-6, and RANTES expression	RELMβ expression was associated with ileitis initiation, but causal involvement was not established by genetic or selective blocking experiments	Associative mouse evidence: limited	(69)
Rodent HIMF/RELMα and human RELMβ *in vitro*	Pulmonary hypertension	HIMF is induced in hypoxic rodent lungs; human RELMβ was examined in pulmonary artery smooth muscle cells	Chronic-hypoxia and Sugen/hypoxia rat models; cultured human pulmonary artery smooth muscle cells	BMPR1A–BMPR2 disruption and suppression of SMAD1/5/9–ID1 signaling	*In vivo* evidence supports a causal role for rodent HIMF. Corresponding effects of human RELMβ were demonstrated mainly in cultured cells and do not establish full mechanistic equivalence	Direct rodent HIMF evidence: moderate; inference for human RELMβ: limited	(16)
Human RELMβ	Systemic-sclerosis-associated pulmonary arterial hypertension and pulmonary vascular remodeling	RELMβ is increased in remodeled vessels, plexiform lesions, and perivascular inflammatory cells; cultured vascular cells respond to recombinant RELMβ	Human lung specimens and cultured pulmonary vascular cells	ERK1/2, FAK–survivin, PLC–KCNK3, and TGF-β1–SMAD2/3/4 pathways	Human tissue findings establish disease association, while cultured-cell studies demonstrate proliferative and phenotypic effects; causal activity in patients has not been established.	Human association plus direct *in vitro* evidence: moderate; no *in vivo* causality	(17–19, 29)
Rodent RELMβ and recombinant human RELMβ in rodent models	Experimental hypoxic or monocrotaline-induced pulmonary hypertension	RELMβ is increased in hypoxic lung tissue and pulmonary artery smooth muscle cells	Hypoxia- or monocrotaline-induced rodent models; *Retnlb* deletion, silencing, and pharmacological intervention	CaSR–PLC–IP_3_R, PI3K–AKT–mTOR, PKC–MAPK, MFN2-dependent mitochondrial fission, and PINK1–Parkin mitophagy	RELMβ promoted smooth muscle-cell proliferation and vascular remodeling in rodent models. These pathways were identified in separate studies and should not be interpreted as one established signaling cascade.	Direct genetic and interventional rodent evidence: moderate to strong within these models	(20–22, 33, 34)
Human and mouse RELMβ	COPD-associated airway inflammation	RELMβ is increased in COPD lung tissue and bronchial epithelial models and is induced by cigarette-smoke exposure	Human COPD specimens, cigarette-smoke-extract-treated epithelial cells, and cigarette-smoke-exposed mice	NF-κB/p65, p38 MAPK, and autophagy-associated signaling	RELMβ promoted inflammatory mediator production in cell and mouse models; human evidence remains predominantly associative	Human association supported by cell and mouse studies: moderate	(23, 31, 71, 72)
Human and mouse RELMβ	Pulmonary fibrosis	RELMβ is increased in lungs from patients with idiopathic pulmonary fibrosis and strongly induced in bleomycin-treated mice	Human IPF lung tissue, bleomycin-induced fibrosis, and *Retnlb*-deficient mice	IL-4/IL-13–STAT6-associated induction and extracellular-matrix regulation	*Retnlb* deficiency attenuated fibrosis in mice, whereas human data establish expression association rather than causality	Direct genetic mouse evidence: moderate to strong; human evidence remains associative	(74)
Human and mouse RELMβ	Asthma and airway remodeling	RELMβ is increased in asthmatic bronchial tissue and bronchoalveolar lavage fluid and is induced by allergens and type 2 cytokines in mice	Human bronchial specimens and cultured airway cells; allergen-challenged and *Retnlb*-deficient mice	IL-4/IL-13–STAT6, ERK/MAPK, PI3K–AKT, and TGF-β-associated pathways	Human studies support association and direct activity in cultured cells. Mouse studies indicate contributions to acute inflammation, whereas chronic remodeling phenotypes vary with the model and disease stage	Mixed experimental and human associative evidence: moderate but heterogeneous	(55, 75–77, 79)
Rat and mouse RELMβ	Intestinal glucose transport and hepatic insulin resistance	Luminal RELMβ acts on intestinal epithelium, whereas circulating or experimentally overexpressed RELMβ acts on the liver	Rat jejunal transport assays, systemic protein infusion, and RELMβ-transgenic mice	PKC/AMPK-dependent GLUT2 recruitment; ERK/p38 activation; suppression of IRS–PI3K–AKT signaling	RELMβ altered intestinal glucose transport and impaired hepatic insulin action in rodent models; the contribution of these mechanisms to human metabolic disease is unknown	Direct rodent intervention and gain-of-function evidence: moderate	(80–82)
Mouse RELMβ	High-fat-diet-associated metabolic dysfunction	Loss of intestinal RELMβ alters intestinal homeostasis and systemic metabolic phenotypes	*Retnlb*-deficient mice on different genetic backgrounds and dietary regimens	Changes in energy expenditure, intestinal integrity, inflammation, and microbiota composition	Loss-of-function studies produced divergent effects on weight gain and glucose tolerance, indicating strong dependence on genetic background, diet, and intestinal condition	Direct genetic mouse evidence: moderate but inconsistent across models	(83, 84)
Mouse RELMβ	Experimental steatohepatitis	RELMβ is increased in the colon and expressed by hepatic Kupffer cells	Methionine- and choline-deficient diet, *Retnlb*-deficient mice, and bone-marrow chimeras	Circulating LPS, inflammatory signaling, and TIMP-1-associated matrix remodeling	Both hematopoietic and non-hematopoietic RELMβ contributed to steatosis, inflammation, and fibrosis in this model. The model does not reproduce the obesity and insulin resistance characteristic of human MASH	Direct genetic and bone-marrow-chimera evidence: moderate to strong within this model	(24)
Human and mouse RELMβ	Atherosclerosis	RELMβ is abundant in macrophage-derived foam cells in human plaques and experimental lesions	Human atherosclerotic tissue, primary macrophages, and *Retnlb*^−/−^*Apoe*^−/−^ mice	NF-κB-associated inflammation and regulation of lipid transporters and scavenger receptors	Human tissue establishes plaque association, while genetic mouse experiments support a contribution to foam-cell formation and lesion development	Human association plus direct genetic mouse evidence: moderate to strong	(25)
Human and mouse RELMβ	Abdominal aortic aneurysm	Circulating and tissue RELMβ are increased in human AAA and in angiotensin-II-induced mouse aneurysms	Human samples and angiotensin-II-treated *Apoe*^−/−^ mice with RELMβ silencing	ERK1/2 and JNK activation; induction of MCP-1, IL-6, MMP-2, and MMP-9	RELMβ silencing reduced aneurysm formation in mice; human evidence remains associative and does not establish the source of vascular RELMβ	Human association plus interventional mouse evidence: moderate.	(26, 37)
Human RELMβ	Hyperglycemia-associated vascular remodeling	RELMβ augments high-glucose-induced phenotypic changes in human aortic smooth muscle cells	High-glucose-treated human aortic smooth muscle cells	Smooth muscle-cell phenotypic modulation and migration-associated signaling	The study demonstrates activity in cultured human cells but does not establish that RELMβ independently drives diabetic atherosclerosis *in vivo*	Direct *in vitro* evidence only: limited	(85)

At the luminal surface, recombinant murine and human RELMβ permeabilized susceptible bacterial membranes, while murine *Retnlb* restricted bacterial penetration into the inner colonic mucus layer (8). Murine RELMβ also supported barrier-associated responses: *Retnlb*-deficient mice showed increased basal epithelial permeability and reduced colonic RegIIIβ and RegIIIγ expression, and developed more severe TNBS-induced colitis than wild-type mice (12). Consistently, intracolonic administration of recombinant RELMβ increased mucus secretion and attenuated TNBS-induced injury (13). During *Citrobacter rodentium* infection, murine RELMβ promoted CD4^+^ T-cell recruitment, IL-22 production, and epithelial proliferation (11). Recombinant RELMβ also increased *MUC2* and M1/MUC5AC expression and secretion in a human goblet-like colonic epithelial cell line (13), although this epithelial cell-line finding has not been validated directly in patients with IBD.

By contrast, two murine studies independently found that *Retnlb* deficiency reduced the severity of DSS-induced colitis (10, 12). In one study, recombinant murine RELMβ induced TNF-α and IL-15 production in isolated macrophages, while intraperitoneal administration increased macrophage TNF-α expression and neutrophil recruitment (10). These experiments demonstrate myeloid-cell activation but do not establish that luminal RELMβ crosses the damaged epithelium and directly activates lamina propria macrophages. In the SAMP1/YitFc model of spontaneous Crohn-like ileitis, ileal RELMβ expression increased with disease onset and histological injury, providing associative rather than causal evidence (69). Stronger genetic evidence was obtained in *Muc2*-deficient mice, in which additional *Retnlb* deletion attenuated spontaneous colitis, reduced RegIIIβ production, and preserved colonic *Lactobacillus* populations (14).

Taken together, murine RELMβ supports antimicrobial and epithelial-repair responses in some settings but promotes inflammation or microbiome disruption in others. These differences are established across distinct experimental models; they do not yet demonstrate that epithelial barrier disruption itself causes a switch in RELMβ activity. Direct evidence linking RELMβ to human IBD pathogenesis remains limited.

### 4.2 Pulmonary inflammation and remodeling

#### 4.2.1 Pulmonary hypertension and vascular remodeling

Evidence from human tissues, cultured vascular cells, and experimental pulmonary hypertension models links RELMβ to pulmonary vascular remodeling, although the strength of this evidence differs across systems. In lung specimens from patients with systemic sclerosis-associated pulmonary arterial hypertension, RELMβ was increased and localized to remodeled vessels, plexiform lesions, and perivascular inflammatory cells (17). These findings establish an association with human disease but do not demonstrate that RELMβ directly drives vascular remodeling *in vivo*. Moreover, the cellular and anatomical origin of disease-associated RELMβ has not been systematically determined, because tissue staining alone cannot distinguish local production from uptake of circulating protein.

Direct functional evidence for human RELMβ is derived mainly from cultured pulmonary vascular cells. Recombinant human RELMβ promoted human pulmonary artery smooth muscle cell proliferation through FAK-associated induction of survivin, whereas FAK silencing partially reduced this response (18). In cultured human endothelial cells, TGF-β1 induced *RETNLB* transcription through SMAD2/3/4, and RELMβ contributed to endothelial-to-mesenchymal transition (29). In hypoxic human pulmonary artery smooth muscle cells, increased RELMβ was also associated with PLC-dependent loss of membrane KCNK3, while RELMβ silencing or PLC inhibition attenuated KCNK3 reduction and cell proliferation (19). These studies indicate that human RELMβ can promote proliferative and phenotypic changes relevant to vascular remodeling, but the evidence remains predominantly *in vitro*, and the physiological relevance of the recombinant protein concentrations used has not been fully established.

Complementary preclinical studies have linked RELMβ-associated signaling to intracellular Ca²^+^ elevation, PI3K–AKT–mTOR and PKC–MAPK activation, MFN2-dependent mitochondrial fragmentation, and PINK1–Parkin-mediated mitophagy (20–22). More recently, *Retnlb* knockout in rats attenuated hypoxia-induced elevation of right ventricular systolic pressure, right ventricular hypertrophy, pulmonary arterial thickening, and pulmonary artery smooth muscle cell proliferation (33). Co-immunoprecipitation, colocalization, and CaSR inhibition implicated CaSR-associated PLC–IP_3_R–Ca²^+^ signaling, although direct binding kinetics were not determined (33). An earlier study similarly identified CaSR as a nonclassical signaling partner for murine HIMF/RELMα in experimental pulmonary hypertension (70), but whether CaSR recognition is conserved across the RELM family remains unresolved. These processes converge on pulmonary artery smooth muscle cell proliferation and vascular remodeling, although they were identified in different models and should not be interpreted as a single established signaling cascade. Rodent HIMF/RELMα has been used as a context-specific functional comparator for human RELMβ in pulmonary vascular research because both proteins are hypoxia responsive and exert mitogenic and remodeling-associated effects. Beyond the CaSR-related findings, HIMF/RELMα was shown to bind BMPR1A and disrupt BMPR1A–BMPR2 signaling in rodent pulmonary hypertension models, whereas evidence for recombinant human RELMβ was limited to supplementary experiments in cultured human pulmonary artery smooth muscle cells (16). These findings support a disease-specific functional comparison but do not establish sequence orthology or mechanistic equivalence outside the pulmonary vascular context (16).

Overall, human data associate RELMβ with pulmonary vascular remodeling and demonstrate direct activity in cultured vascular cells, whereas stronger causal evidence is currently derived from experimental models involving rodent RELMβ or its pulmonary vascular functional analogue HIMF/RELMα. Further human *in vivo* evidence is required before RELMβ can be regarded as an established driver or therapeutic target in pulmonary arterial hypertension.

#### 4.2.2 Chronic obstructive pulmonary disease

In COPD, clinical and experimental evidence links RELMβ primarily to airway inflammation. RELMβ expression is increased in lung tissue and serum from patients with COPD and has been associated with impaired pulmonary function, disease severity, and increased expression of IL-8 and IL-1β (23, 31). In a human bronchial epithelial cell line, cigarette smoke extract induced RELMβ expression, whereas RELMβ overexpression or recombinant human RELMβ increased inflammatory cytokine production through p38 MAPK and NF-κB/p65 signaling (31, 71). Complementary experiments using a tobacco smoke- and elastase-induced murine COPD-like model showed that *Retnlb* deficiency attenuated airway inflammation and autophagy-associated changes (72). Thus, human studies demonstrate an association between RELMβ and COPD severity, while direct evidence for its contribution to airway inflammation is derived mainly from epithelial-cell experiments and mouse models.

#### 4.2.3 Acute pulmonary infection

Elevated circulating RELMβ has also been reported in acute pulmonary infection. In a multicenter cohort of patients with community-acquired pneumonia, serum RELMβ concentrations were higher in severe disease and were associated with clinical severity scores and 30-day mortality (73). This finding supports an association between circulating RELMβ and the severity of acute pulmonary inflammation but does not establish its cellular source or causal role.

#### 4.2.4 Pulmonary fibrosis

RELMβ has also been implicated in pulmonary fibrosis, although the evidence again differs between species. RELMβ expression was increased in lung tissue from patients with idiopathic pulmonary fibrosis, providing evidence of an association with human fibrotic disease (74). In bleomycin-treated mice, pulmonary RELMβ was induced by type 2 cytokines through STAT6, and *Retnlb* deficiency reduced collagen deposition, profibrotic cytokine expression, and overall fibrosis severity (74). RELMβ also promoted collagen and α-smooth muscle actin expression in cultured lung fibroblasts. Together, these findings support a causal profibrotic role for murine RELMβ in experimental pulmonary fibrosis, whereas its contribution to human idiopathic pulmonary fibrosis remains supported mainly by tissue-expression data.

#### 4.2.5 Asthma

Current evidence links RELMβ to airway inflammation and remodeling in asthma but does not support a uniformly pathogenic or protective role. In patients with asthma, RELMβ was increased in bronchial mucosa and bronchoalveolar lavage fluid, and its local expression was associated with lower pulmonary function and increased mucin production (75, 76). Bronchoconstriction also induced RELMβ expression in asthmatic airways (75). *In vitro*, recombinant human RELMβ promoted airway epithelial-cell proliferation and mucin production and stimulated human lung fibroblast proliferation, myofibroblast differentiation, and extracellular matrix production (76, 77). These findings support a potential contribution to human airway remodeling, although direct causality has not been demonstrated. A separate, relatively small clinical study reported lower serum RELMβ concentrations in patients with asthma (78), indicating that circulating concentrations do not necessarily reflect local airway expression.

Murine studies have produced less consistent functional results. Allergens and IL-4/IL-13 induced pulmonary RELMβ through STAT6-dependent signaling, while intratracheal administration of recombinant murine RELMβ caused leukocyte accumulation, goblet-cell hyperplasia, and collagen deposition (55). In an *Aspergillus fumigatus* model, *Retnlb* deficiency reduced the expression of selected extracellular matrix components (77). By contrast, a separate chronic *A. fumigatus* model showed that *Retnlb*-deficient mice had less tissue inflammation but greater goblet-cell hyperplasia, airway remodeling, and airway hyperresponsiveness (79). Thus, the consequences of RELMβ deficiency vary among murine asthma models and experimental stages, and its role in human asthma remains supported primarily by tissue associations and cell-based remodeling effects.

### 4.3 Hepatic and metabolic disorders

Evidence linking RELMβ to glucose and lipid metabolism is derived predominantly from rodent studies. In high-fat-fed and obese *db/db* mice, increased circulating RELMβ was accompanied by increased production in the colon and bone marrow (35). At the intestinal level, luminal recombinant RELMβ increased glucose transport across rat jejunum by reducing SGLT-1 activity while promoting PKC- and AMPK-dependent recruitment of GLUT2 to the apical membrane (80). This experiment established a direct effect on intestinal glucose transport but did not determine its contribution to postprandial hyperglycemia *in vivo*. Systemic infusion of recombinant RELMβ in rats selectively impaired hepatic insulin action by increasing hepatic glucose production, without measurably reducing peripheral glucose disposal (81).

Longer-term metabolic effects have been examined in transgenic mice with hepatic RELMβ overexpression. When fed a high-fat diet, these mice developed more pronounced hyperglycemia, hyperlipidemia, and hepatic steatosis (82). In primary hepatocytes and transgenic mice, RELMβ activated ERK and p38 MAPK and reduced IRS-1/IRS-2 abundance and insulin-stimulated PI3K–AKT signaling (82). These findings support a direct inhibitory effect on hepatic insulin signaling in this model but do not establish the same mechanism in human metabolic disease.

Whole-body loss-of-function studies, however, have not demonstrated a uniformly protective metabolic phenotype. In one cohort, *Retnlb*-deficient mice gained less weight and fat mass during high-fat feeding despite similar food intake and fat absorption, an incompletely penetrant phenotype associated with increased oxygen consumption (83). By contrast, a separate study using *Retnlb*-deficient mice on C57BL/6 and FVB backgrounds reported impaired glucose tolerance even without dietary challenge or obesity, accompanied by intestinal abnormalities and inflammation in mesenteric adipose tissue (84). These divergent findings indicate that the metabolic consequences of RELMβ vary among experimental models and that its circulating effects on hepatic insulin action should be distinguished from its local role in intestinal homeostasis.

The strongest evidence for a role in steatohepatitis comes from a methionine- and choline-deficient diet model. *Retnlb-*deficient mice developed less steatosis, inflammation, and fibrosis than wild-type mice (24). Bone-marrow chimera experiments further indicated that both non-hematopoietic RELMβ, including colonic RELMβ, and hematopoietic RELMβ expressed by Kupffer cells contributed to the full hepatic phenotype. Loss of either source was associated with lower circulating LPS and reduced hepatic inflammatory and fibrotic responses (24). However, because this dietary model causes weight loss and does not reproduce the obesity or insulin resistance characteristic of human metabolic dysfunction-associated steatohepatitis (MASH), these findings should be interpreted as evidence from experimental steatohepatitis rather than direct validation of a mechanism in human metabolic liver disease.

Overall, rodent studies show that RELMβ can influence metabolism through distinct intestinal and hepatic pathways, with whole-body *Retnlb* deficiency and excess circulating or hepatic RELMβ producing different outcomes. Whether either mechanism contributes to human MASH remains unknown.

### 4.4 Cardiovascular diseases

Current evidence links RELMβ to macrophage-rich vascular lesions, although human and experimental studies provide different levels of mechanistic support. In human coronary atherosclerotic plaques, RELMβ was detected predominantly in foam cells, establishing its local association with vascular inflammation (25). In *Apoe*^−/−^ mice, additional loss of *Retnlb* reduced aortic lipid accumulation without significantly altering circulating lipid concentrations. Complementary experiments in mouse macrophages linked RELMβ to NF-κB-dependent inflammatory responses and the expression of proteins involved in lipid uptake and foam-cell formation (25). In cultured human aortic smooth muscle cells, recombinant RELMβ also enhanced high-glucose-induced phenotypic switching, migration, and proliferation, partly through p38 and ERK signaling (85). These findings implicate both macrophages and vascular smooth muscle cells in RELMβ-associated vascular changes, but direct evidence that RELMβ drives human atherosclerosis remains unavailable.

RELMβ has also been associated with abdominal aortic aneurysm (AAA). A clinical study reported increased RELMβ expression in human aneurysmal aortic tissue and higher circulating concentrations in patients with AAA (37). In Ang II–infused *Apoe*^−/−^ mice, systemic siRNA-mediated *Retnlb* knockdown reduced AAA incidence and severity, macrophage infiltration, MCP-1 and IL-6 expression, and MMP-2 and MMP-9 abundance (26). In mouse macrophages, recombinant RELMβ activated ERK1/2 and JNK, whereas inhibition of these pathways attenuated the induction of inflammatory mediators and MMPs (26). Thus, RELMβ is associated with human atherosclerotic and aneurysmal lesions, whereas evidence for a causal role in lesion progression remains predominantly preclinical.

## 5 RELMβ in digestive tract cancers

RELMβ expression is increased in oral squamous cell carcinoma (OSCC) compared with adjacent non-tumor tissues, and higher *RETNLB* expression has been associated with shorter overall survival. *RETNLB* knockdown suppressed OSCC cell viability, migration, and invasion, possibly through modulation of the TLR2/4–ERK signaling pathway (86). However, these findings require further clinical validation.

In the stomach, RELMβ is largely absent from normal mucosa but is detected in intestinal metaplasia and a subset of gastric cancers. RELMβ positivity has been associated with intestinal-type differentiation, less invasive disease, and longer survival (87). However, overexpression studies in different human gastric cancer cell lines yielded opposing results, showing either suppression of invasion and angiogenesis or promotion of migration, invasion, and EMT (88, 89). Aberrant gastric RELMβ expression may be partly induced by *Helicobacter pylori* through its *cag* pathogenicity island, consistent with the association between RELMβ expression and *H. pylori* density in clinical specimens (90). Thus, the functional significance of RELMβ in gastric cancer remains uncertain.

Studies using transcriptomic and immunohistochemical analyses have reported different patterns of RELMβ expression in colorectal cancer (91, 92). Nevertheless, lower or absent expression was associated with poorer survival in both studies. Overall, evidence for RELMβ in digestive tract cancers remains limited and inconsistent across tumor types and experimental systems.

## 6 Future directions

As a cysteine-rich secretory protein of the RELM family, RELMβ functions at the interface of host defense, mucosal immunity, and metabolic regulation. Several biological features are central to understanding its context-dependent activities: (i) human RELMβ, encoded by *RETNLB*, is a small secreted protein with a molecular weight of approximately 8.5 kDa. Its precursor comprises approximately 111 amino acids, while the mature secreted form generated after signal peptide cleavage contains approximately 88 amino acids. Although mature human RELMβ shares approximately 48.5% sequence similarity with human resistin, the two proteins differ in chromosomal location, predominant cellular source, and reported biological activity (93). (ii) Like other RELM-family proteins, RELMβ possesses a modular architecture with an N-terminal α-helical “tail” and a conserved cysteine-rich C-terminal β-sandwich “head” domain (32, 48). (iii) RELMβ is predominantly expressed in intestinal goblet cells, while inducible expression in extraintestinal tissues and selected immune cells has been reported under inflammatory and pathological conditions (17, 24, 25, 35); and (iv) its biological effects vary across anatomical and pathological settings, encompassing mucosal defense, immune regulation, and metabolic signaling. For RELMβ, anatomical compartment, target accessibility, and local physicochemical conditions may shape its effects, but their relative contributions are not yet established.

### 6.1 Context-dependent receptor engagement

RELMβ-responsive cells have been identified in the intestine and pulmonary vasculature, but receptor studies remain concentrated in pulmonary vascular models. In rodent hypoxia-associated pulmonary hypertension, HIMF/RELMα was reported to bind BMPR1A, disrupt the BMPR1A–BMPR2 complex, and suppress downstream SMAD1/5/9–ID1 signaling (16). Supplementary *in vitro* data from the same study suggested that recombinant human RELMβ associates with BMPR1A and perturbs the BMPR1A-BMPR2 signaling complex in human pulmonary artery smooth muscle cells (16). Thus, the BMPR1A–BMPR2 complex represents a plausible pulmonary vascular interaction system for HIMF and potentially human RELMβ, although the evidence for human RELMβ remains less established than that for murine HIMF in rodent pulmonary hypertension models. Moreover, whereas hypoxia induces local pulmonary HIMF expression in rodents, it remains unknown whether RELMβ associated with pulmonary vascular disease is derived predominantly from local vascular or inflammatory cells or from gut-derived protein entering the circulation.

Within the pulmonary vascular compartment, RELMβ may engage more than one molecular system. A recent study reported co-immunoprecipitation and colocalization of RELMβ with CaSR in pulmonary artery smooth muscle cells, while pharmacological CaSR inhibition attenuated RELMβ-associated PLC–IP_3_R signaling, Ca²^+^ mobilization, and cell proliferation (33). This finding extends an earlier study in which murine HIMF/RELMα acted as a nonclassical CaSR ligand in experimental pulmonary hypertension (70). The shared structural organization of RELM-family proteins raises the possibility of a conserved basis for CaSR recognition, but no common binding motif or comparative binding analysis across family members has been reported. CaSR should therefore be regarded as a plausible context-specific receptor or signaling partner for RELMβ rather than a universal RELM-family receptor. Separately, affinity purification–mass spectrometry (AP–MS) and co-immunoprecipitation identified GIPC1, OR1N1, and CLIC4 as RELMβ-associated proteins (34). OR1N1 was the only GPCR among these candidates, but direct ligand-binding kinetics and *in vivo* genetic evidence were not provided. Thus, OR1N1 remains a putative receptor, whereas GIPC1 and CLIC4 are more appropriately classified as associated signaling proteins. More broadly, phospholipid–chemoattractant signaling illustrates how GPCR responses can be shaped by membrane lipid organization and convergence through PLC–IP_3_–Ca²^+^ and PI3K–AKT pathways that regulate immune-cell migration and inflammatory activity (94). This framework may inform studies of compartment-specific RELMβ responsiveness, but it does not establish a RELMβ-specific lipid–chemoattractant pathway. Overall, these findings support multiple candidate interaction systems in hypoxic pulmonary vascular cells but do not yet establish a coordinated or universally applicable receptor ensemble.

Outside the pulmonary vasculature, RELMβ-responsive cells have been identified, but their receptors remain unknown. In mouse and human intestinal organoids, exogenous RELMβ increased endogenous *Retnlb/RETNLB* expression and cooperated with IL-13, indicating an epithelial response through an unidentified sensor (15). The *Heligmosomoides polygyrus bakeri* protein HpBoRB binds murine RELMβ with subnanomolar affinity (66), but it is a parasite-derived binding protein rather than a host receptor, and the functional consequences remain unclear.

Human resistin has been reported to signal through CAP1 and TLR4 (45, 95–97), but these findings cannot be directly extended to RELMβ. Supplementary *in vitro* data suggested a RELMβ–CAP1 interaction, whereas RELMβ-induced macrophage activation and foam-cell formation have not been shown to depend on CAP1 (25, 45). Across these studies, interaction sites have been mapped more precisely on candidate partners—the intracellular domain of CaSR, the extracellular DTLPF motif of BMPR1A, and the proline-rich SH3-binding region of CAP1—than on the RELM ligands themselves (16, 45, 70). In the CAP1 study, predicted resistin-contacting residues were localized within the C-terminal globular region of resistin by molecular docking but were not validated by ligand-side truncation or mutagenesis (45). This receptor–ligand domain-mapping asymmetry may partly reflect the small size, disulfide-rich architecture, and assembly-dependent behavior of RELM-family proteins. Their folding, oligomerization, and accessible binding surfaces may be altered by truncation, mutation, or fusion tags, making direct mapping of the RELM-side interface technically difficult. Any receptor-binding contribution of the RELMβ C-terminal region would be mechanistically distinct from the demonstrated antibacterial membrane activity of the murine RELMβ C-terminal region.

More recent evidence implicates the resistin–CAP1 axis in intermediate-monocyte–T-cell communication during liver allograft rejection, indicating that CAP1-dependent resistin activity extends beyond the original monocyte model (98). Together, these studies suggest that CAP1 contributes to human resistin signaling in more than one disease context but provide no basis for assuming the same interaction across other RELM-family members. Separately, human resistin can compete with LPS for TLR4 binding and attenuate endotoxin-induced inflammation (99), illustrating that engagement of an identified interaction partner does not necessarily produce a uniform biological outcome. Thus, current evidence supports context-specific candidate interactions rather than a universal or defined cell type–specific receptor ensemble.

### 6.2 Domain-specific membrane activity

RELMβ provides the clearest evidence for direct membrane-disruptive activity within the RELM family. Murine RELMβ preferentially binds negatively charged lipids and permeabilizes bacterial membranes, with greater activity against the Gram-negative bacteria tested. In liposome assays, its isolated C-terminal region retained lipid-binding and membrane-permeabilizing activity, whereas the N-terminal peptide showed little activity. Membrane-associated cross-linking yielded an approximately 60–70-kDa complex, consistent with an estimated assembly of six to eight subunits, and dye-release experiments supported the formation of size-selective membrane lesions (8). Full-length human resistin also permeabilized bacterial and PC/PS liposome membranes and formed membrane-associated multimers, indicating that membrane-disruptive activity is shared by at least some RELM-family proteins (8). However, comparison of full-length human resistin with the murine RELMβ C-terminal fragment does not establish that the C terminus constitutes a conserved membrane-disrupting domain throughout the family.

RELMβ-mediated bactericidal and membrane-permeabilizing activities were enhanced under acidic, low-ionic-strength conditions *in vitro (*8). These findings indicate that its membrane activity is strongly influenced by the local physicochemical environment. Nevertheless, whether these conditions spatially restrict RELMβ activity within the intestinal mucus layer or protect host epithelial membranes from collateral damage has not been demonstrated directly.

Recent findings on murine RELMγ extend RELM-family membrane activity from microbial targets to damaged host cells. Under acidic conditions, RELMγ accumulated at cardiomyocyte membrane regions displaying externalized phosphatidylserine, formed dense carpet-like deposits, and produced membrane defects associated with delayed afterdepolarizations and cell injury (46). The authors proposed that electrostatic attraction to negatively charged membrane lipids is followed, after a local concentration threshold is reached, by insertion of a hydrophobic α-helix into the lipid bilayer. Unlike the multimeric pore model proposed for RELMβ, this carpet mechanism does not require formation of a discrete oligomeric pore. Moreover, because the contributions of isolated RELMγ domains were not tested directly, a specific “C-terminal anchoring–N-terminal execution” mechanism remains hypothetical rather than established.

Pore-forming or pore-like membrane activity has been demonstrated for several RELM-family members. Murine RELMα, murine and human RELMβ, and human resistin permeabilize bacterial or bacterial-mimetic membranes (8, 40), whereas murine RELMγ and human resistin produce pore-like defects in phosphatidylserine-containing membranes and damaged host cells (46). These findings suggest that pore-forming or pore-like membrane activity recurs across several RELM-family members, although the underlying molecular mechanisms may differ. RELMβ membrane permeabilization is primarily associated with its C-terminal region and a membrane-bound multimeric complex, whereas the proposed RELMγ carpet model emphasizes electrostatic membrane association and hydrophobic α-helical insertion. Acidic conditions may facilitate both processes, but should not yet be regarded as a universal activation switch. Determining whether different RELM proteins use common structural elements in distinct combinations—or employ genuinely different modes of membrane recognition, oligomerization, and disruption—will require domain-specific mutagenesis and structural analysis of intact proteins on physiologically relevant membranes (**Figure 4**).

**Figure 4:**
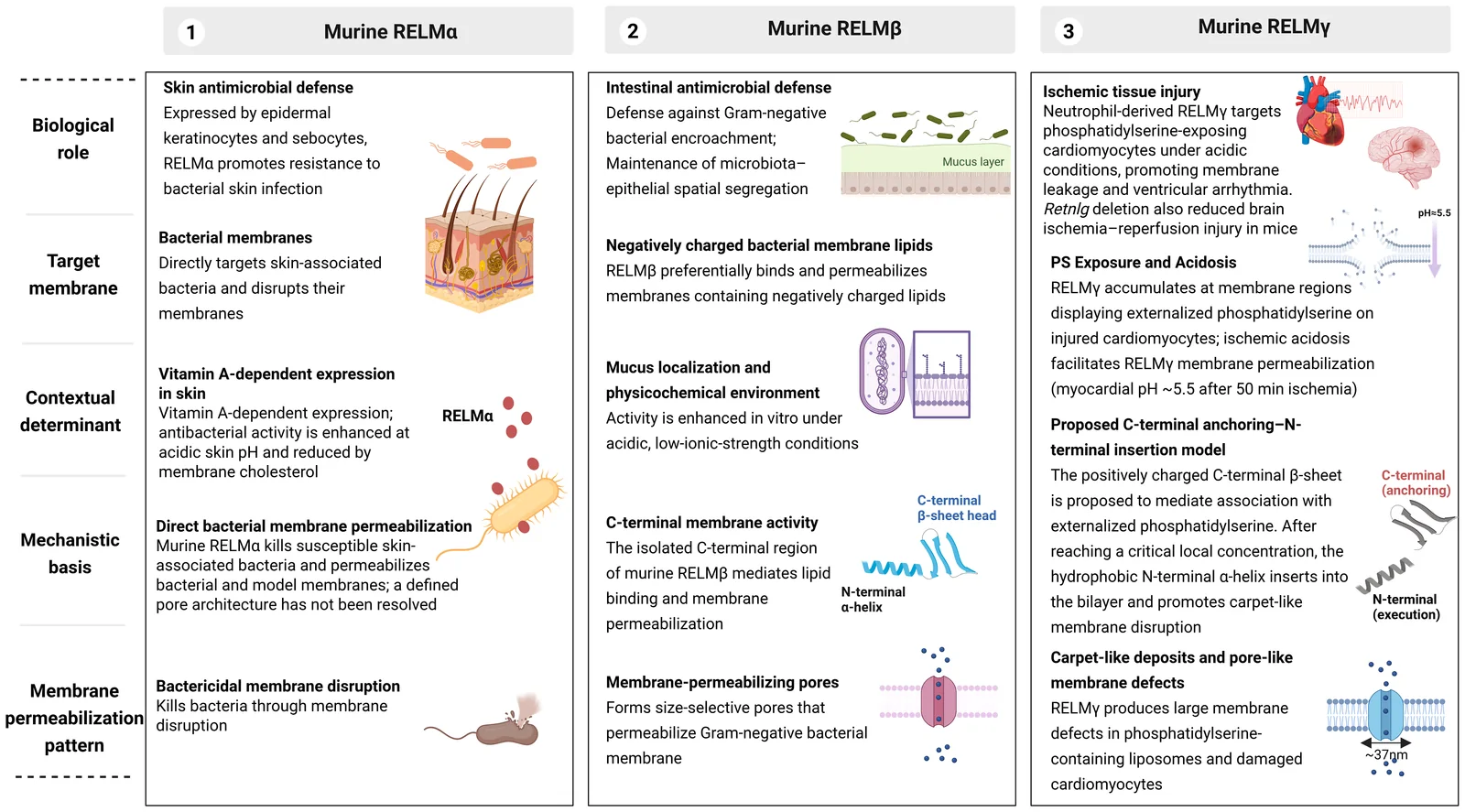
Context-dependent pore-forming and membrane-disruptive activities across the RELM family. Comparison of the biological settings, target membranes, contextual determinants, proposed mechanisms, and membrane damage patterns reported for murine RELMα, RELMβ, and RELMγ. The mechanisms shown are based on different experimental systems and should not be interpreted as a single conserved pore-forming mechanism across the RELM family. PS, phosphatidylserine. Created in BioRender. ai, Q. (2026) https://BioRender.com/jak2wr2.

### 6.3 Limitations and experimental priorities

Interpretation of current evidence is limited by uncertainty regarding RELMβ-specific receptors, compartment-specific concentrations, and native oligomeric states in human tissues and plasma. Additional limitations include potential artifacts from recombinant-protein experiments, heterogeneity among animal models, and limited causal evidence in humans. Moreover, mechanisms established for murine RELMα/HIMF, RELMγ, or human resistin cannot be directly assigned to human RELMβ. Accordingly, RELMβ has not been validated as a clinical biomarker, therapeutic target, or patient-stratification tool.

Future studies should first establish the cellular and anatomical origin of RELMβ detected in pathological tissues. It may arise from locally induced expression, basolateral secretion by intestinal epithelial cells, or entry of intestine-derived RELMβ into the circulation after barrier disruption. Because immunostaining and plasma ELISA cannot distinguish among these possibilities, cell type–specific genetic manipulation or endogenous protein tracing should be combined with parallel analysis of the intestinal lumen, lamina propria, circulation, and diseased tissues. These approaches could test whether barrier disruption increases extraintestinal exposure to intestine-derived RELMβ and shifts its activity from luminal antimicrobial defense toward immune or vascular effects. Cross-species interpretation must also account for the lineage-specific expansion of murine *Retnl* genes, which precludes assigning the function of a particular murine RELM protein directly to a human family member based only on nomenclature or phenotypic similarity.

A second priority is to determine whether anatomical compartmentalization alters RELMβ interaction partners, molecular assembly, and biological outcome. No universal receptor has been established for RELMβ, and the relevant interaction system may vary with receptor availability, RELMβ concentration and oligomeric state, pH, ionic strength, lipid composition, and local binding partners. The motif mutation and blocking-peptide approaches used to investigate HIMF–BMPR1A interactions provide a useful experimental framework, but corresponding interactions must be validated independently with human RELMβ and human cells. If pathological RELMβ activity depends on a tissue-enriched interaction system, selective disruption of that interaction should reduce inflammation or vascular remodeling without impairing luminal bactericidal activity, microbiota–epithelial segregation, or defense against susceptible lumen-dwelling nematodes. This prediction should be tested through genetic perturbation or interaction-selective blockade, accompanied by parallel assessment of diseased.

Overall, three priorities warrant further investigation to clarify the pathological and therapeutic significance of RELMβ. First, its receptor-mediated effects require more rigorous characterization. The identification of several candidate interaction systems suggests that RELMβ recognition may vary among cell types and disease settings rather than depend on a universal receptor. This question is particularly relevant to RELMβ-associated metabolic and vascular conditions. Second, receptor-mediated signaling must be distinguished from direct membrane activity. Studies of RELMβ and other RELM-family proteins support membrane-disruptive activity in selected contexts, but do not establish a conserved pore-forming mechanism, common domain requirement, or uniform lipid specificity across the family. Finally, the cellular origin of pathological RELMβ remains unresolved. Determining whether RELMβ detected in vascular or pulmonary tissues arises from local paracrine production, intestine-derived protein entering the circulation, or both will help establish whether context-selective interventions can suppress pathological activity without compromising its antimicrobial and barrier-protective functions in the intestinal lumen.

## 7 Conclusion

RELMβ, predominantly secreted by intestinal goblet cells, is positioned at the interface between mucosal immunity and host–microbiota homeostasis. Its established antibacterial activity at the intestinal mucosal surface contrasts with its reported proinflammatory and tissue-remodeling activities in intestinal and extraintestinal disease settings, although the mechanisms underlying these distinct functions remain incompletely understood. The proposed compartment-interaction system framework provides a basis for organizing these observations but remains a testable hypothesis, because direct evidence linking RELMβ redistribution to altered interaction-partner engagement is currently lacking.
